# Novel C5α-substituted carbapenems enhance *Mycobacterium abscessus* killing via selective target binding and reduced hydrolysis by Bla_Mab_

**DOI:** 10.1128/aac.00170-25

**Published:** 2025-06-17

**Authors:** Eunjeong Shin, Magdalena A. Taracila, Pojun Quan, Md Mahbub Kabir Khan, Jonathan Cox, Diya Patel, Khalid M. Dousa, Aetan Parmar, Mary Nantongo, David C. Nguyen, Eric J. Rubin, Steven M. Holland, Barry N. Kreiswirth, John D. Buynak, Robert A. Bonomo

**Affiliations:** 1 Louis Stokes Cleveland VA Medical Center, Case Western Reserve University 2546, Cleveland, Ohio, USA; 2 Department of Medicine, Case Western Reserve University School of Medicine 12304, Cleveland, Ohio, USA; 3 Department of Chemistry, Southern Methodist University 2765, Dallas, Texas, USA; 4 Ohio State University 2647, Columbus, Ohio, USA; 5 Department of Molecular Biology and Microbiology, Case Western Reserve University School of Medicine 12304, Cleveland, Ohio, USA; 6 Department of Pediatrics, Division of Infectious Diseases, Rush University Medical Center 2468, Chicago, Illinois, USA; 7 Department of Internal Medicine, Division of Infectious Diseases, Rush University Medical Center, Chicago, Illinois, USA; 8 Department of Immunology and Infectious Diseases, Harvard T.H. Chan School of Public Health 1857 , Boston, Massachusetts, USA; 9 Laboratory of Clinical Immunology and Microbiology, National Institute of Allergy and Infectious Diseases, National Institutes of Health 35037, Bethesda, Maryland, USA; 10 Center for Discovery and Innovation, Hackensack Meridian Health, Nutley, New Jersey, USA; 11 CWRU-Cleveland VAMC Center for Antibiotic Resistance and Epidemiology (Case VA CARES), Cleveland, Ohio, USA; 12 Department of Biochemistry, CWRU 2546 , Cleveland, Ohio, USA; 13 Department of Pharmacology, CWRU 2546 , Cleveland, Ohio, USA; 14 Department of Proteomics and Bioinformatics, CWRU 2546 , Cleveland, Ohio, USA; 15 Department of Pharmacology, Case Western Reserve University School of Medicine 12304, Cleveland, Ohio, USA; 16 Department of Proteomics and Bioinformatics, Case Western Reserve University School of Medicine 12304, Cleveland, Ohio, USA; 17 Cleveland Geriatrics Research Education and Clinical Center (GRECC), VANEOHS 273136 , Cleveland, Ohio, USA; City St George's, University of London, London, United Kingdom

**Keywords:** *Mycobacterium abscessus*, carbapenems, L,D-transpeptidases, penicillin-binding proteins

## Abstract

*Mycobacterium abscessus* (*Mab*) poses significant clinical challenges and underscores the urgent need for safer and more effective treatments, including β-lactams. Among currently available carbapenems, imipenem is widely used to treat *Mab* infections by combining with other antibiotics. Commercial carbapenems share a common scaffold with C2 modifications, whereas this study focuses on novel carbapenem candidates with C5α modifications. We evaluated their antibacterial activity against *Mab* ATCC 19977 and clinical isolates, as well as their acylation of peptidoglycan target receptors (L,D-transpeptidases [LDTs] and penicillin-binding proteins [PBPs]) and the β-lactamase enzyme Bla_Mab_. *In vitro* studies of two C5α-modified carbapenems, JDB/NA-1-157 and JDB/NA-1-208, revealed distinct antibacterial effects. JDB/NA-1-157 demonstrated potent bacterial killing with low minimum inhibitory concentrations (MICs; 0.125–8 mg/L) and near-complete eradication within 5 days, surpassing the efficacy of the standard-of-care regimen (amikacin + clarithromycin + imipenem). In contrast, JDB/NA-1-208 exhibited poor bacterial killing, with high MICs (16–256 mg/L) and limited efficacy in time-kill studies. However, JDB/NA-1-208 showed synergistic killing when combined with other β-lactams. Mechanistically, JDB/NA-1-208 is not a substrate for Bla_Mab_, while JDB/NA-1-157 is, albeit with low catalytic efficiency. This is supported by the observation that the addition of avibactam did not enhance synergy with JDB/NA-1-157. The substantial bacterial killing effect of JDB/NA-1-157 is attributed to its high binding affinity for PBP-B, PBP-lipo, PonA2, D,D-carboxypeptidase, and LDT1–2. These findings highlight the potential of novel C5α-modified carbapenems, particularly JDB/NA-1-157, as promising therapeutic candidates for *Mab* infections.

## INTRODUCTION

*Mycobacterium abscessus* (*Mab*), a nontuberculous mycobacterium, poses substantial challenges in clinical settings (1) due to its innate resistance to first-line antimycobacterial agents and most currently available antibiotics. This pathogen is one of the major causes of chronic pulmonary infections, particularly in individuals with compromised immune systems or underlying structural lung conditions, such as bronchiectasis and cystic fibrosis, and is associated with significant mortality (2). Resistance to macrolides, mediated by the induction or acquisition of resistance genes such as *erm*(41) and *rrl*, contributes to failure rates as high as ~70% (3, 4). These rates exceed those reported for multidrug-resistant tuberculosis and extensively drug-resistant tuberculosis (5, 6). Current treatments for *Mab* infections demonstrate high toxicity, primarily due to amikacin-related adverse effects (7–9) and poor outcomes (10), underscoring the urgent need for more effective therapeutic strategies.

Although β-lactams are included in the treatment regimen for *Mab*, they have never been used as monotherapy, and their efficacy against *Mab* remains uncertain due to the production of Bla_Mab_, an endogenous serine class A β-lactamase. Bla_Mab_ is efficiently inhibited by newer inhibitors, including diazabicyclooctanes (e.g., avibactam, relebactam, nacubactam, zidebactam, and durlobactam) and boronic acid derivatives (e.g., vaborbactam) (11–19). Combinations of β-lactams with other β-lactams or β-lactamase inhibitors (BLIs) have demonstrated promising antimycobacterial activity (15, 16, 19–25).

In *Mab*, β-lactams exert their antibacterial effects by targeting both L,D-transpeptidases (LDTs) and D,D-transpeptidases (also known as penicillin-binding proteins [PBPs]) (26). Disruption of LDT and PBP activity interferes with key processes in cell wall biosynthesis, maturation, and remodeling, ultimately leading to bacterial death. However, comprehensive binding data for β-lactams and BLIs targeting *Mab*’s PBPs and LDTs remain scarce, with only limited reports available in the literature (20, 25, 27). Limited studies suggest that LDT1, LDT2, PBP-lipo, D,D-carboxypeptidase (DDC), and PBP B may play roles in peptidoglycan synthesis and bacterial viability in *Mab* (25, 28–30).

Most carbapenems currently in use are based on modifications to the C2 position of the core carbapenem scaffold. However, the emergence of novel carbapenemases across multiple pathogens has significantly undermined their efficacy. As part of efforts to combat carbapenemase-mediated hydrolysis and resistance, the Buynak group has explored the development of atypical carbapenems substituted at positions other than C2, such as C5α-substituted carbapenems (e.g., C5α-methyl and C5α-ethyl derivatives) (31). These atypical modifications have the potential to alter the interactions of carbapenems with β-lactamases and LDTs/PBPs. Specifically, they may influence key properties such as noncovalent binding, the rate of acylation of target proteins, and, for β-lactamases, the rate of acyl-enzyme hydrolysis, potentially offering a novel strategy to overcome resistance mechanisms.

**Fig 1:**
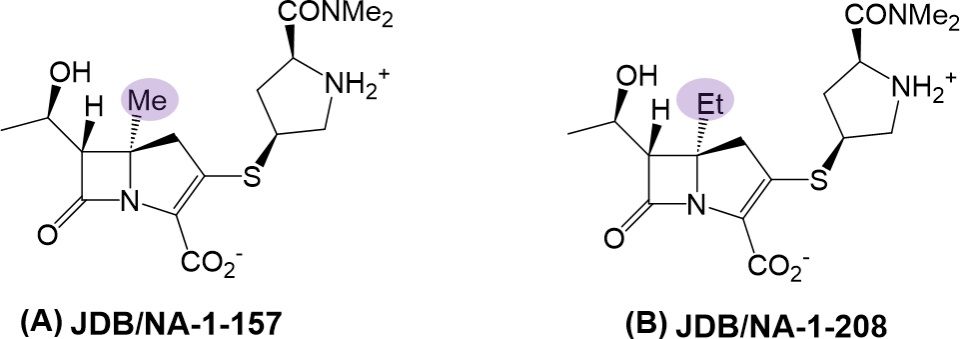
Chemical structures of (A) JDB/NA-1-157 and (B) JDB/NA-1-208.

Here, this study aimed to assess the antibacterial activity of two C5α-substituted carbapenems, JDB/NA-1-157 and JDB/NA-1-208 (Fig. 1), against *Mab*, both as stand-alone and in combination with other β-lactams or BLIs. Additionally, the investigation explored the ability to bind target receptors (LDTs and PBPs) and correlated bacterial killing efficacy with target protein binding affinity. The potential susceptibility of these carbapenems to Bla_Mab_-mediated hydrolysis was further examined using mass spectrometry and kinetic studies, providing critical insights into their biochemical interactions and stability.

## RESULTS

### Stability in 7H9 broth at 30°C

**Fig 2:**
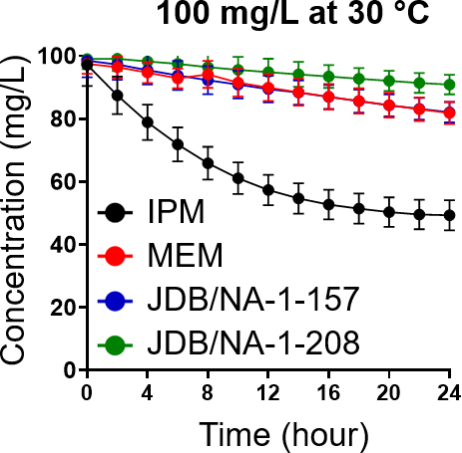
Thermal stability of imipenem (IPM), meropenem (MEM), JDB/NA-1-157, and JDB/NA-1-208 was evaluated at 100 mg/L in 7H9 broth at 30°C over 24 h. JDB/NA-1-208 demonstrated the highest thermal stability, with approximately 10% degradation observed after 24 h. JDB/NA-1-157 exhibited 16% degradation over the same period.

Imipenem (IPM), meropenem (MEM), and the two C5α-substituted carbapenem candidates (JDB/NA-1-157 and JDB/NA-1-208) exhibited varying degrees of instability (Fig. 2; Fig. S2 and Table S1). Compared to imipenem, three compounds (meropenem, JDB/NA-1-157, and JDB/NA-1-208) were more stable, with 10%–15% degradation observed over 24 h. JDB/NA-1-208 exhibited the longest half-life (*t*_1/2_) of 66 h, followed by JDB/NA-1-157 with a *t*_1/2_ of 50 h. In contrast, imipenem was markedly unstable, with a *t*_1/2_ of only 10 h. To evaluate the effect of β-lactam instability, we performed minimum inhibitory concentration (MIC) tests on the four carbapenems after 6, 24, and 48 h of incubation (i.e., after expected degradation) against *Mab* ATCC 19977 (Table S2). While no MIC change was observed after 6 h of incubation compared to nonincubated controls, after 24 and 48 h incubation, MICs increased significantly. Meropenem and JDB/NA-1-157 (15% degradation at 24 h) showed a fourfold MIC rise. JDB/NA-1-208, the most stable, had a twofold shift, while imipenem, the least stable, exhibited 32-fold and >64-fold increases.

### *In vitro* antimycobacterial activity

**Fig 3:**
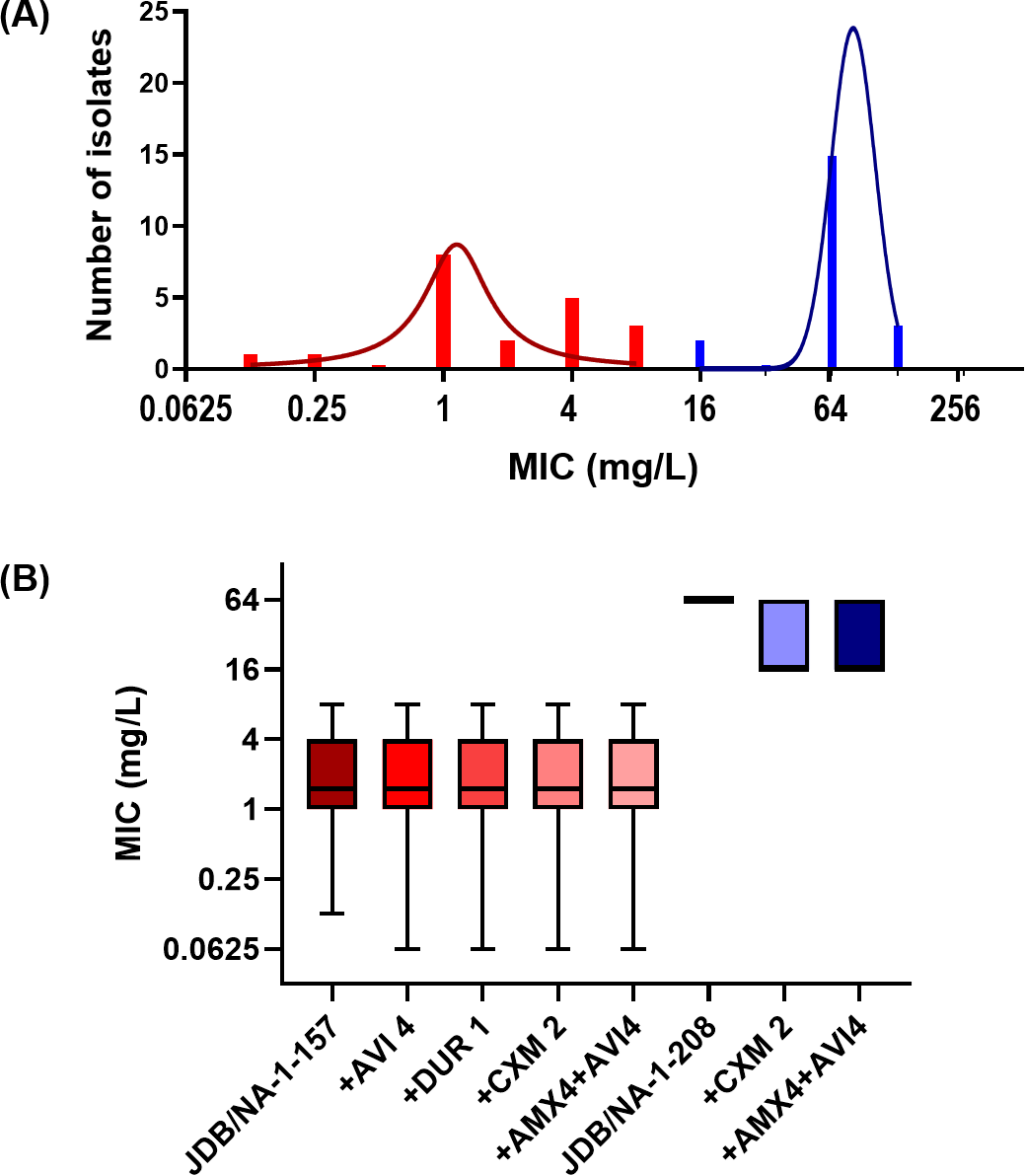
MIC distribution of JDB/NA-1-157 and JDB/NA-1-208 as single agent or in combination against 18 clinical isolates and *Mab* ATCC 19977. (**A**) JDB/NA-1-157 (red) demonstrated MIC values ranging from 0.125 to 8.0 mg/L, while JDB/NA-1-208 (blue) exhibited significantly higher MIC values, ranging from 16 to >128 mg/L. (**B**) JDB/NA-1-157, when combined with avibactam (AVI) 4 mg/L, durlobactam (DUR) 1 mg/L, cefuroxime (CXM) 2 mg/L, or amoxicillin (AMX) 4 mg/L + AVI 4 mg/L, resulted in a slight reduction in MIC values, though they remained similar to monotherapy. JDB/NA-1-208, in combination with CXM 2 mg/L or AMX 4 mg/L + AVI 4 mg/L, showed a decrease in MIC for some isolates compared to single JDB/NA-1-208.

The antibacterial activity of two atypical C5α-substituted carbapenems (JDB/NA-1-157 and JDB/NA-1-208) was assessed against *Mab*, both as a single agent and in combination with cefuroxime, ceftaroline, amoxicillin, avibactam, and durlobactam. For MIC testing, although JDB/NA-1-208 exhibited high MIC values when tested alone (16 to >128 mg/L), JDB/NA-1-157 demonstrated potent antibacterial activity, with a low MIC of 0.5 mg/L against *Mab* ATCC 19977 (Table 1) and 0.125–8.0 mg/L in 18 clinical isolates (Fig. 3), consistent with findings from the Buynak group (31). Compared to imipenem, the most potent carbapenem against *Mab* (20, 25), the MIC of JDB/NA-1-57 alone is remarkably low, indicating a strong antibacterial effect. The addition of avibactam or durlobactam led to slight changes (one- to twofold increases) in the MICs of JDB/NA-1-157, while the MIC of JDB/NA-1-208 remained unchanged. In contrast, combining JDB/NA-1-157 with cefuroxime, ceftaroline, or amoxicillin (with avibactam) resulted in up to a 4-fold reduction in MIC and up to a 4- to ≥1,000-fold decrease for JDB/NA-1-208 (Table 1; Fig. 3).

**TABLE 1:** Minimum inhibitory concentrations of JDB/NA-1-157 and JDB/NA-1-208 were determined against the *Mab* ATCC 19977 strain, both as a single agent and in combination with AVI 4 mg/L, DUR 1 mg/L, CXM 2 mg/L, CFT 4 mg/L, and AMX 4 mg/L + AVI 4 mg/L^*a*^

	JDB/NA-1-157	JDB/NA-1-208	No carbapenem
Alone	0.5	32	
+AVI (4 mg/L)	0.5	32	>128
+DUR (1 mg/L)	0.25	32	8
+CXM (2 mg/L)	0.125	8	4
+CFT (4 mg/L)	0.125	4	16
+AMX (4 mg/L) + AVI (4 mg/L)	0.125	≤0.0313	Without AVI: 128, with AVI: 32

^*a*^ AMX, amoxicillin; AVI, avibactam; CFT, ceftaroline; CXM, cefuroxime; DUR, durlobactam.

**Fig 4:**
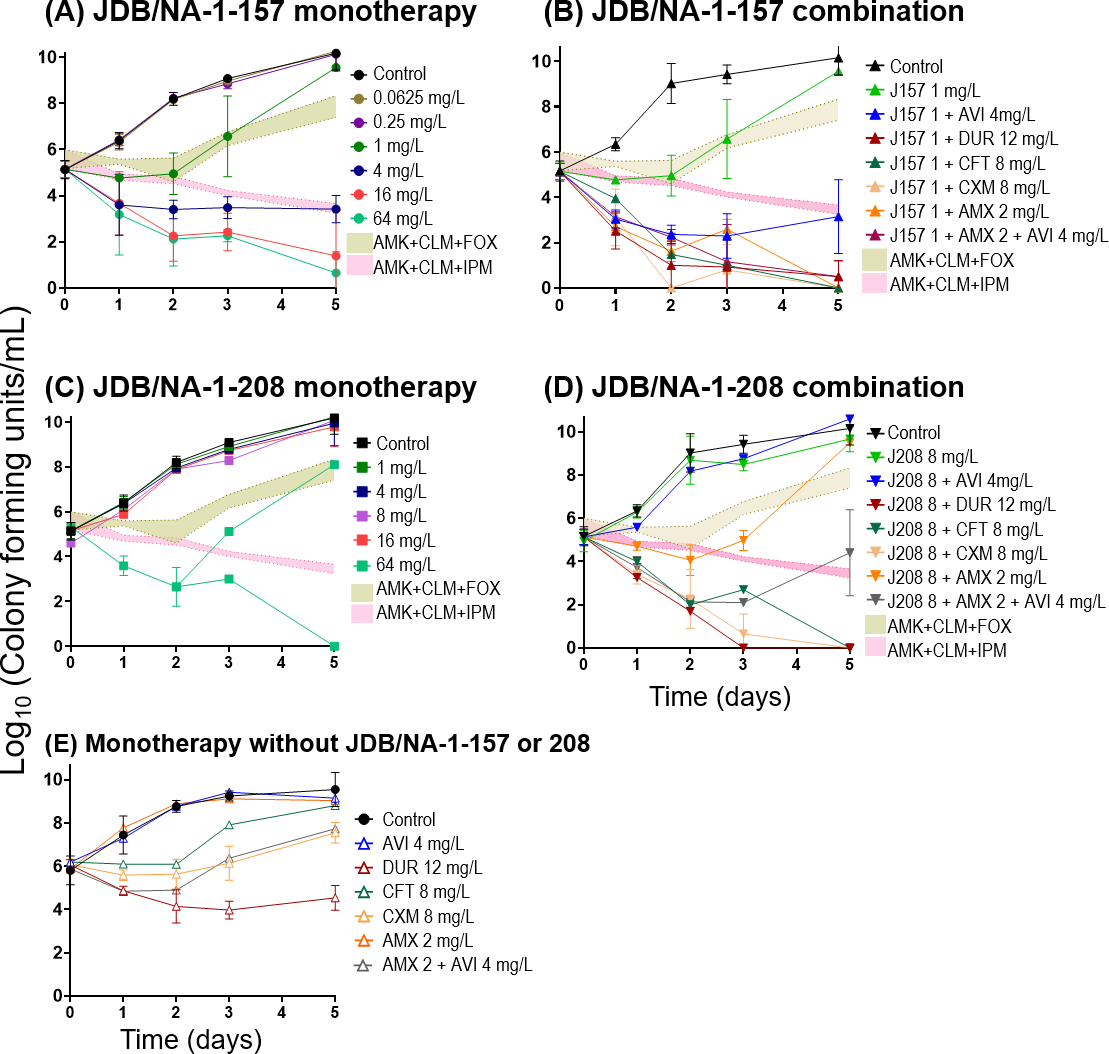
Time-kill curves of JDB/NA-1-157 as a single agent (**A**); in combination with β-lactamases, cephalosporins, or penicillin (**B**); JDB/NA-1-208 as a single agent (**C**); and in combination with β-lactamases, cephalosporins, or penicillin (**D**) against Mab ATCC 19977. (**E**) Time-kill curves without JDB/NA-1-157 or JDB/NA-1-208, demonstrating the effects of avibactam (AVI) 4 mg/L, durlobactam (DUR) 12 mg/L, ceftaroline (CFT) 8 mg/L, cefuroxime (CXM) 8 mg/L, amoxicillin (AMX) 2 mg/L, and AMX 2 mg/L + AVI 4 mg/L. Standard-of-care regimens, including amikacin (AMK) 12 mg/L + clarithromycin (CLM) 0.3 mg/L + cefoxitin (FOX) 7 mg/L or AMK 12 mg/L + CLM 0.3 mg/L + imipenem (IPM) 12 mg/L, were used as comparators to assess the efficacy of JDB/NA-1-157 and JDB/NA-1-208.

**Fig 5:**
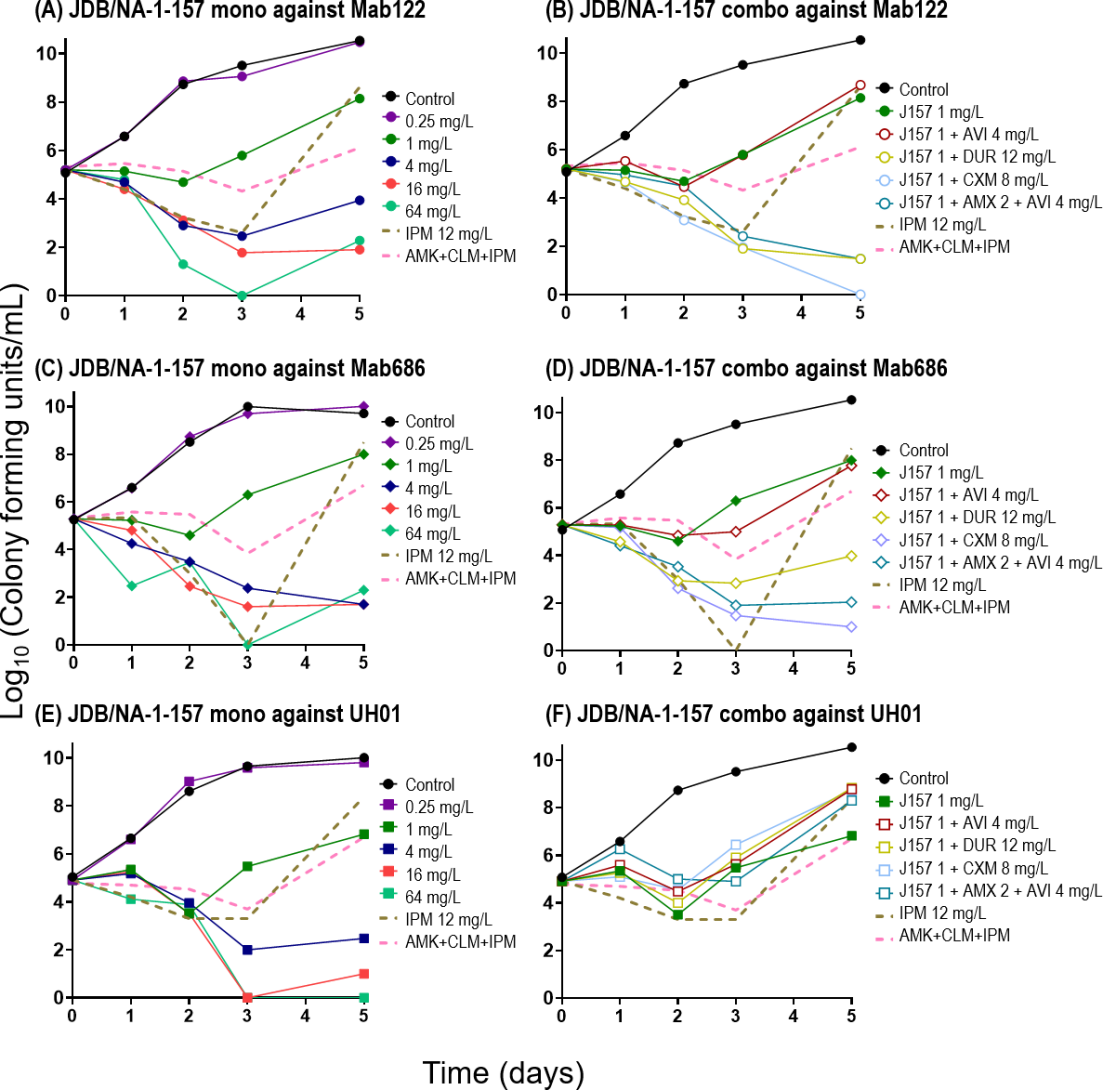
The efficacy of JDB/NA-1-157 and JDB/NA-1-208, either as a single agent or in combination with β-lactamases, cephalosporins, or penicillin, was evaluated using time-kill assay against selected clinical isolates Mab122 (**A and B**), Mab 686 (**C and D**), and UH01 (**E and F**).

A similar antibacterial activity of JDB/NA-1–157 and JDB/NA-1-208 was observed in time-kill assays (Fig. 4 and 5). JDB/NA-1-157 alone demonstrated potent bactericidal activity against ATCC 19977 and the clinical isolates, with detectable effects at a low concentration of 1 mg/L, achieving a 1.5–2.0 log_10_ CFU/mL reduction at 4 mg/L over 5 days, and near-complete eradication at concentrations of 16–64 mg/L (Fig. 4A; Fig. 5A, C, and E), superior to imipenem and the standard-of-care (SOC) regimen (amikacin + clarithromycin + imipenem). In combination with BLIs (avibactam or durlobactam) or β-lactams (cefuroxime, ceftaroline, or amoxicillin), JDB/NA-1-157 produced significant bacterial killing, with near-complete eradication observed by day 3 in ATCC 19977 and the two clinical isolates (Mab122 and Mab686). However, in the UH01 isolate, the 1 mg/L concentration of JDB/NA-1-157 appeared insufficient, even when combined with other β-lactams or BLIs, to completely eliminate the bacteria. Additionally, the effect of avibactam in combination with JDB/NA-1-157 varied slightly between ATCC 19977 and the three clinical isolates. In ATCC 19977, the addition of avibactam resulted in bacterial killing of up to 2.5 log_10_ CFU/mL. However, in the clinical isolates, avibactam had minimal or no impact on synergistic bacterial killing, suggesting that JDB/NA-1-157 is a poor substrate for Bla_Mab_.

Conversely, single JDB/NA-1-208 showed poor antibacterial activity, with ~3 log_10_ CFU killing observed at high concentrations (64 mg/L). However, combinations of JDB/NA-1-208 with ceftaroline, cefuroxime, amoxicillin (with avibactam), and durlobactam resulted in marked bacterial killing (near-complete eradication). Combining JDB/NA-1-208 with avibactam had no significant effect, indicating that JDB/NA-1-208 is not a substrate for hydrolysis by Bla_Mab_. In contrast, the combination with durlobactam led to substantial bacterial killing, which may be attributed to durlobactam’s ability to inhibit LDTs and PBPs (25).

### Binding assay to Bla_Mab_, L,D-transpeptidases, and penicillin-binding proteins

**Fig 6:**
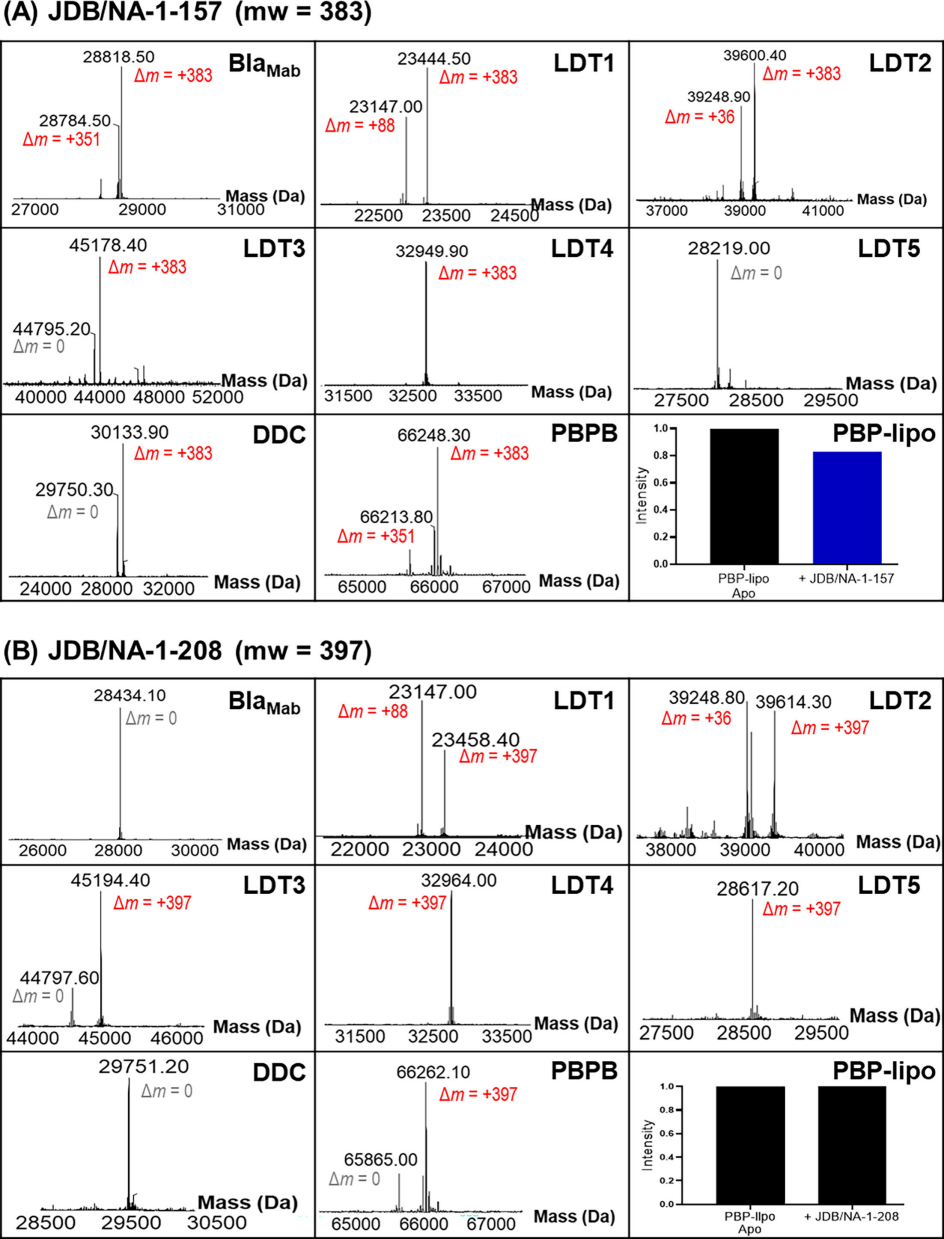
Binding of (A) JDB/NA-1-157 and (B) JDB/NA-1-208 to Bla_Mab_, LDT1–5, D,D-carboxypeptidase, PBP B, and PBP-lipo. The intensities of the PBP-lipo complex bands were quantified using GelAnalyzer software and normalized to the intensity of PBP-lipo without β-lactams (PBP-lipo Apo), which was set to 1.

We assessed the interactions of JDB/NA-1-157 and JDB/NA-1-208 with β-lactamase, Bla_Mab_, and peptidoglycan synthesis enzymes (LDT1–5, DDC, PBP B, and PBP-lipo) (Fig. 6; Table 2) using mass spectrometric (MS) analysis and Bocillin-FL competition assays. JDB/NA-1-157 bound to Bla_Mab_ and formed a complex, while JDB/NA-1-208 did not form an adduct with Bla_Mab_. JDB/NA-1-157 was shown to form acyl-enzyme complexes with LDT1-4, PBP B, DDC, and PBP-lipo. Similarly, JDB/NA-1-208 acylated most LDTs and PBPs, except for DDC and PBP-lipo.

**TABLE 2:** Mass spectrometry analyses of LDTs 1–5, DDC, and PBP B were performed both individually and in the presence of JDB/NA-1-157 or JDB/NA-1-208^*a*^

	JDB/NA-1-157 (mw 383)	JDB/NA-1-208 (mw 397)
Bla_Mab_ (28,433)	28,784 (+351), 28,818 (+383)	28,434 (.)^*b*^
LDT1 (23,059)	23,445 (+383); 23,147 (+88)	23,458 (+397); 23,147 (+88)
LDT2 (39,216)	39,249 (+36); 39,600 (+383)	39,249 (+36); 39,614 (+397)
LDT3 (44,795)	45,178 (+383)	45,194 (+397)
LDT4 (32,567)	32,950 (+383)	32,964 (+397)
LDT5 (28,220)	28,219 (.)	28,617 (+397)
DDC (29,750)	30,134 (+383)	29,750 (.)
PBP B (65,863)	66,248 (+383); 66,214 (+351)	66,262 (+397)

^*a*^ The mass accuracy was within ±5 Da.

^*b*^ (.) indicates no detectable binding.

**Fig 7:**
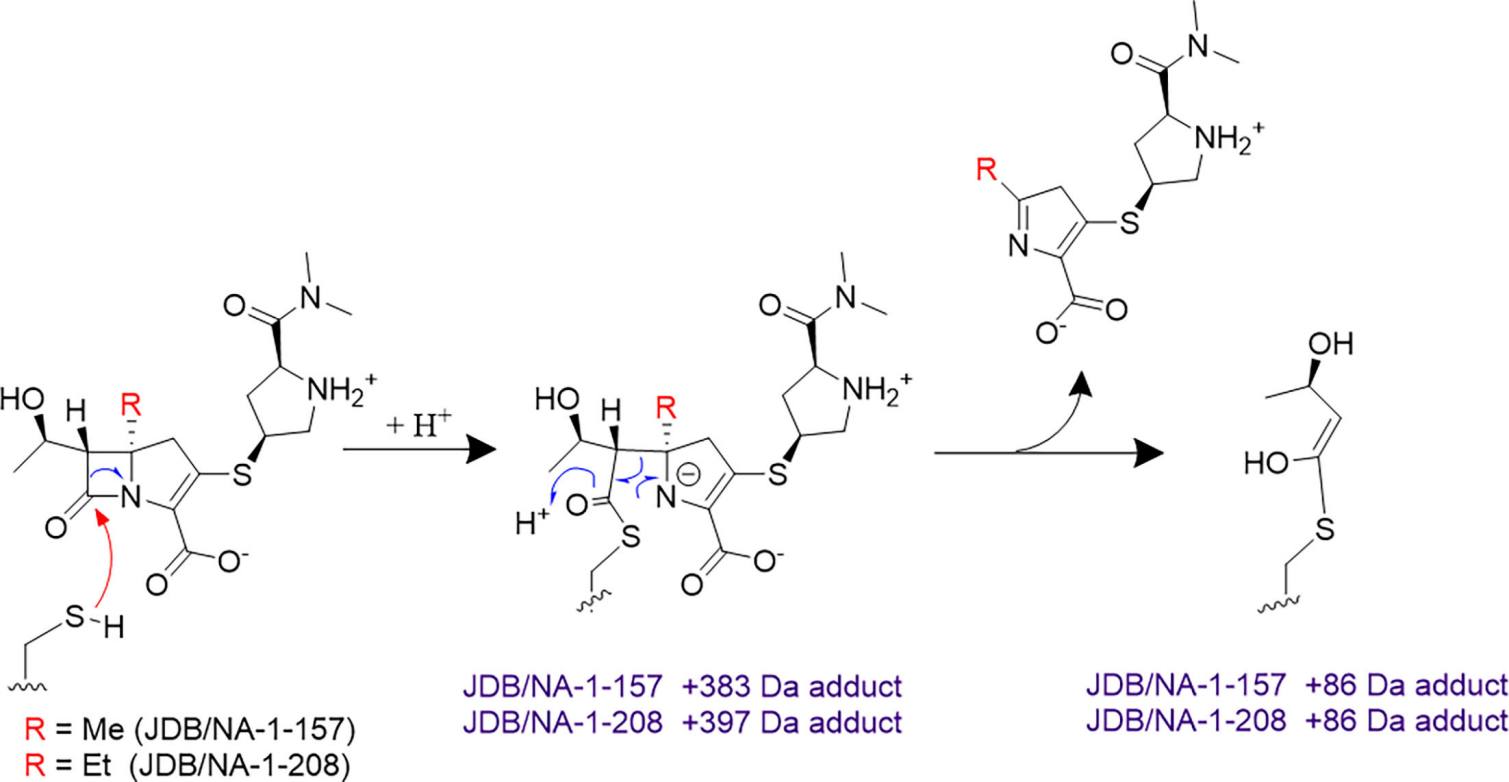
Mechanism of LDT1 acylation by JDB/NA-1-157 and JDB/NA-1-208, followed by the subsequent elimination of the substituent, resulting in an observed Δ*m* = +86 (±5) Da adduct.

Both JDB/NA-1-157 and JDB/NA-1-208 were observed to bind as intact carbapenem antibiotics as well as in fragmented forms to the enzymes. Each exhibited acyl-enzyme complexes with intact compounds and fragments, specifically forming a Δ*m* = +88 Da adduct with LDT1 (Fig. 7). Similar fragmentation adducts have been observed with sulopenem in *Mab* LDT2^16^ or with penem and faropenem in *Mtb* LDT2 (32, 33). Both formed acyl-enzyme complexes with LDT2, binding intact and as fragments, resulting in Δ*m* = +36 Da adducts. JDB/NA-1-157 formed acyl-enzyme complexes as intact molecules only with LDT2-4 and DDC. When bound to Bla_Mab_ and PBP B, JDB/NA-1-157 lost the C6-hydroxyethyl group, yielding a fragmented Δ*m* = +antibiotic – 33 Da adduct. In contrast, JDB/NA-1-208 formed acyl-enzyme complexes only as the intact molecule with LDT2-5 and PBP B.

### How efficiently are JDB/NA-1-157 and JDB/NA-1-208 hydrolyzed by Bla_Mab_, and how effectively do they bind to LDTs and PBPs?

#### Steady-state kinetics of Bla_Mab_ inactivation

The turnover rate (*k*_cat_) and Michaelis constant (*K*_*m*_) values for JDB/NA-1-157 were determined to be 0.03 s^−1^ and 9.054 µM, respectively, yielding a catalytic efficiency of 0.003 µM^−1^·s^−1^ (Table 3). These values indicate that JDB/NA-1-157 is not an effective substrate for hydrolysis by Bla_Mab_ even though it binds to the enzyme.

**TABLE 3:** Kinetic parameters for the hydrolysis of JDB/NA-1-157 by Bla_Mab_ are presented as best-fit values, with the corresponding 95% confidence intervals shown in brackets (determined by profile likelihood)

Compound	*k*_cat_ (s^−1^)	*K*_*m*_ (µM)	*k*_cat_/*K*_*m*_ (µM^−1^·s^−1^)
JDB/NA-1-157	0.030 (0.02733–0.03349)	9.054 (3.677–17.50)	0.003

#### LDT and PBP binding affinities

JDB/NA-1-157 exhibited high to moderate affinity for PBP B, PBP-lipo, DDC, PonA2, and LDT1–2, with <5 mg/L of *K*_i,app_; some targets displayed particularly low *K*_i,app_ (Table 4), with similar or better binding affinity than imipenem and meropenem. Although JDB/NA-1-157 binds to LDT3 and LDT4, the high *K*_i,app_ indicates that it is a poor inhibitor of these enzymes. Our preliminary studies with an LDT3 knockout strain demonstrated that LDT3 does not contribute to bacterial viability in *Mab* (data not shown). Therefore, the poor binding affinity to LDT3 has no impact. Similarly, our preliminary data (not shown) suggest that LDT4 and LDT5 do not play as crucial a role in bacterial viability as LDT3. Therefore, the poor binding of JDB/NA-157 to LDT4 and the lack of binding to LDT5 suggest a selective affinity for essential LDTs and PBPs. JDB/NA-1-208 also showed low *K*_i,app_ for PBP B, PonA2, LDT1–2, and LDT4, indicating high binding affinities for these targets. In contrast, JDB/NA-1-208 exhibited poor binding affinity for LDT3 and LDT5, as evidenced by high *K*_i,app_ values (>10 mg/L).

**TABLE 4:** The apparent inhibition constants (*K*_i,app_) of JDB/NA-1-157 and JDB/NA-1-208 were determined for PBP B, PBP-lipo, D,D-carboxypeptidase, PonA2, and LDTs 1–5^*a*^

*K*_i,app_ (mg/L)	JDB/NA-1-157	JDB/NA-1-208	IPM	MEM
Penicillin-binding proteins				
PBP B	1.88	0.032	0.44	0.37
PBP-lipo	0.099	N/A^*b*^	0.55	N/A
D,D-carboxypeptidase	0.009	N/A	1.3	6.84
PonA2	0.003	0.14	0.94	0.055
L,D-transpeptidases				
LDT1	3.06	0.44	0.002	0.079
LDT2	0.05	0.066	0.002	0.012
LDT3	18.5	12.4	N/A	N/A
LDT4	20.7	0.34	4.2	0.052
LDT5	N/A	11.5	30	N/A

^*a*^ For comparison of binding affinities, the *K*_i,app_ values of the carbapenems imipenem (IPM) and meropenem (MEM) were also determined.

^*b*^ N/A indicates not applicable due to no binding observed.

### Molecular docking of JDB-1-157 into the active site of Bla_Mab_ and as Michaelis–Menten and acyl-enzyme complexes

**Fig 8:**
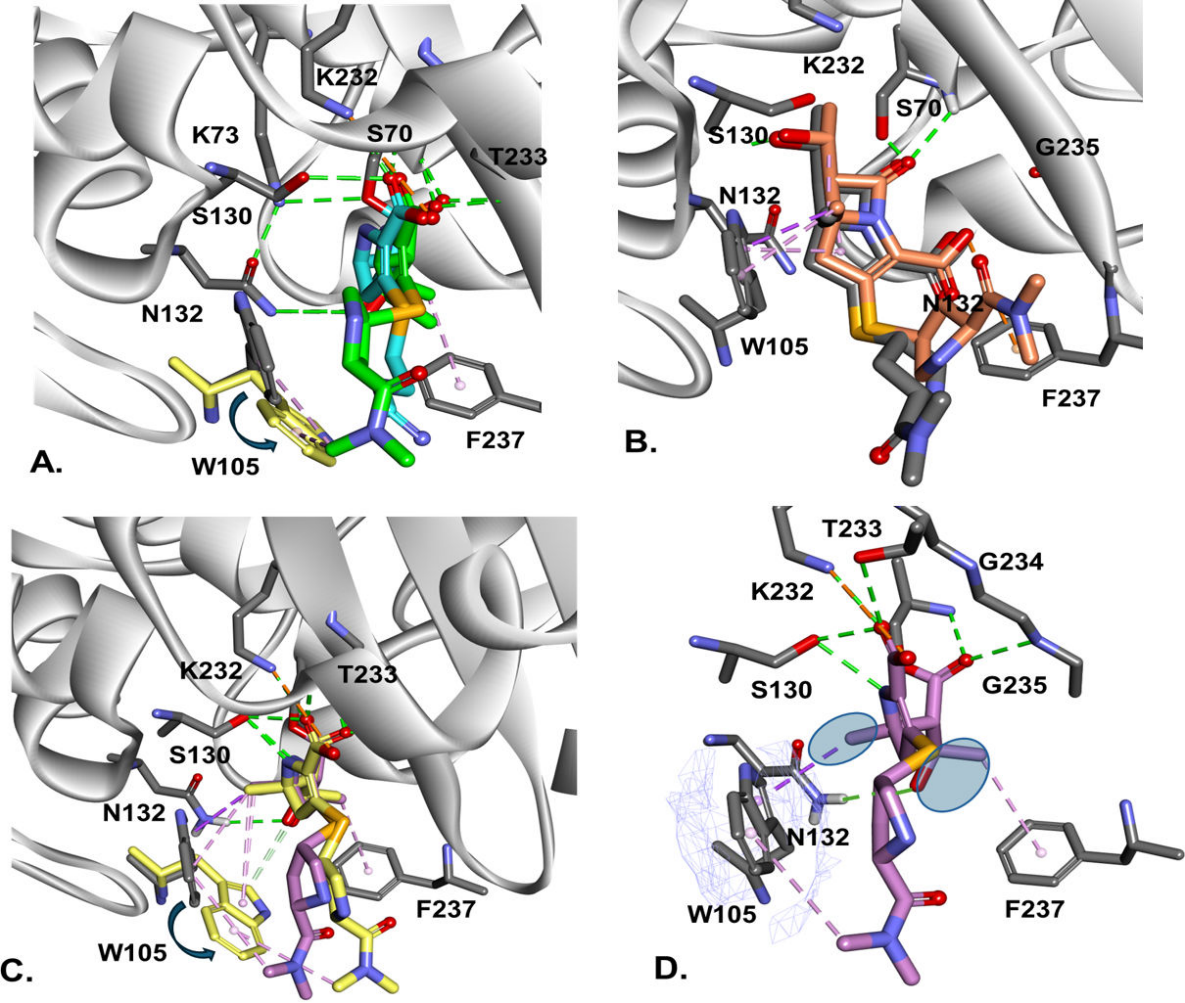
BlaMab with IPM (blue) and MEM (green) as acyl-enzyme complexes (**A**) and the Michaelis–Menten (MM) complex of JDB/NA-1-157 (**B**) show W105 in two conformations (*t-105* “up” and *m-95* “down”). In the MM complex, JDB/NA-1-157 adopts conformations with the carbonyl group oriented toward the oxyanion hole, stabilized by hydrophobic interactions between the indole ring of W105 and a methyl group at C5α. In the acyl-enzyme (**C and D**), the JDB/NA-1-157 core adopts a restrained conformation, with π-methyl stacking interactions with W105 (**C**) and with the hydroxyethyl group interacting with F237 and N132 (**D**). The methyl group maintains its interaction with W105, regardless of its rotamer conformation. Hydrogen bonds with S130, K232, T233, and the oxyanion hole carbonyl are preserved. The JDB/NA-1-157 tail appears more flexible to accommodate conformational changes in the active site.

The Michaelis–Menten complex (Fig. 8B) suggests that JDB/NA-1-157 adopts a conformation where the carbonyl group is oriented toward the oxyanion hole. The core structure of JDB/NA-1-157 appears to be stabilized in this productive conformation, ready for acylation, through hydrophobic interactions between the indole ring of W105 and the newly introduced methyl group at position C5α of JDB/NA-1-157. The pyrrolidine tail seems to be flexible, potentially accommodating multiple rotamer conformations of W105, as observed in the X-ray structure of various class A β-lactamases (34–38). The a.a. at position 105 (W or Y) adopts different conformations (*t-105*, the indole ring up, or *m-95*, the rotamer with the aromatic ring down). In apoenzyme structures, as well as in imipenem and meropenem acyl-enzyme complexes (Fig. 8A), W105 takes the *t-105*-up conformation. In contrast, in other acyl-enzyme structures, such as GES-5 with NA-1-157 (a JDB/NA-1-157 derivative), it shows to be in the *m-95*-down conformation (39).

For the Bla_Mab_-JDB/NA-1-157 acyl-enzyme complex, the W105 rotamers can adopt both conformations (Fig. 8C): *t-105*-up and *m-95* down. In the acyl form, JDB-1-157 adopts a more constrained conformation, with multiple hydrophobic and/or π-methyl stacking interactions involving W105. The core structure of the molecule remains relatively fixed, with the hydroxyethyl moiety interacting hydrophobically with the benzene ring of F237 and forming hydrogen bonds with N132 (Fig. 8D). The newly introduced methyl group maintains its π-methyl interaction with the indole ring of W105, regardless of the rotamer conformation of W105 (Fig. 8C). Additionally, hydrogen bonds are sustained with S130, K232, T233, and the carbonyl group in the oxyanion hole.

**Fig 9:**
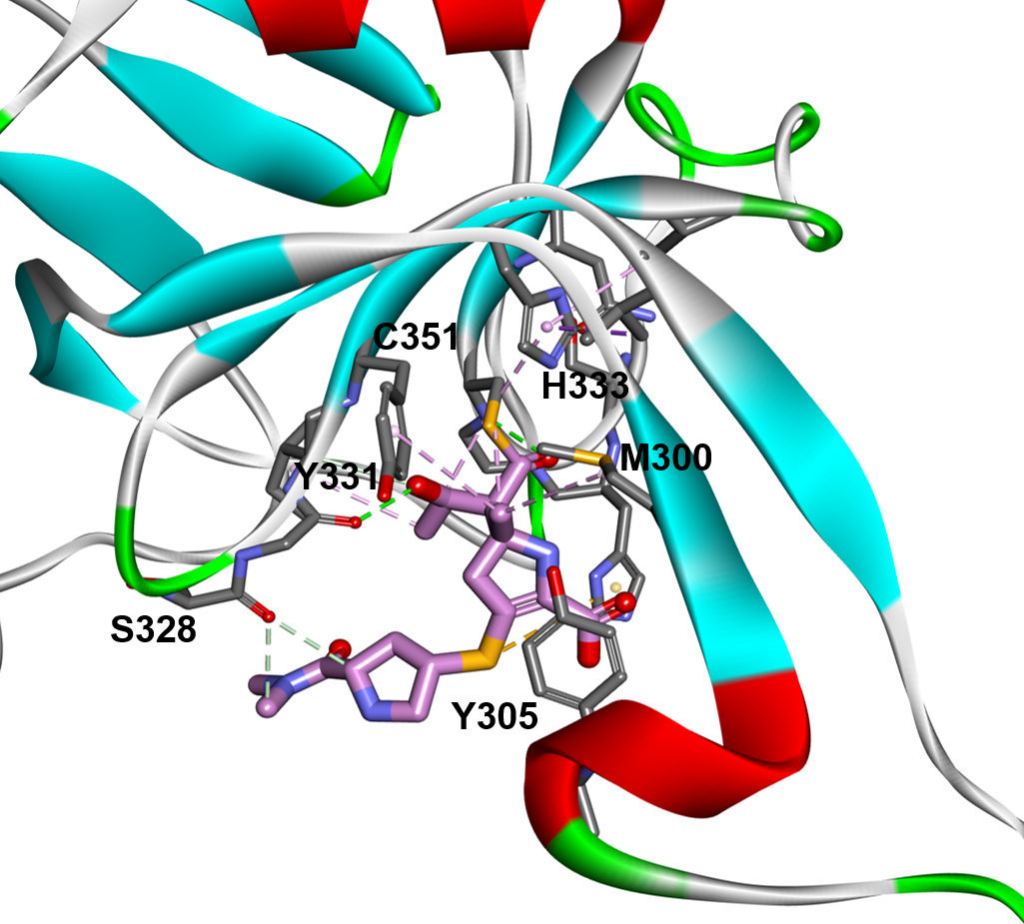
LDT2 and JDB/NA-1-157 acyl-enzyme complex, with JDB/NA-1-157 covalently bound to C351. The carbonyl group forms hydrogen bonds with C351 and G350, while the hydroxyethyl group forms hydrogen bonds with G329. The new C5α methyl group interacts with M300 and Y331, and the tail interacts with S328. Y305, located at the entrance of the active site, may help stabilize the JDB/NA-1-157 compound in a productive conformation through stacking interactions with the pyrrolidine ring.

To explore how JDB/NA-1-157 binds with LDT enzymes, we created and studied the acyl-enzyme complex of LDT2 with JDB/NA-1-157 (Fig. 9). The catalytic cysteine (C351) forms a covalent bond with C7 of JDB/NA-1-157, while the carbonyl group of the compound is positioned into the oxyanion hole, forming hydrogen bonds with C351 and G350. The hydroxyethyl group of JDB/NA-1-157 engages in hydrogen bonding interactions with G329 and exhibits hydrophobic interactions with I330.

The newly introduced methyl group interacts with M300 and Y331, creating hydrophobic contacts. Additionally, the pyrrolidine ring at the tail of JDB/NA-1-157 interacts with S328 (arginine in LDT5) and forms π-sulfur interactions with the conserved H349. Y305, located at the entrance of the active site, may help stabilize the JDB/NA-1-157 compound in a productive conformation through stacking interactions with the pyrroline ring (Fig. 9). The residues N353 (serine in LDT5) and V354 (leucine in LDT5) help anchor the H333 ring, positioning it to form hydrophobic interactions with JDB/NA-1-157.

## DISCUSSION

*Mab* is notoriously challenging to treat (1), with high treatment failure and poor outcomes. Current guidelines include amikacin, which is associated with toxicity (7–9). Furthermore, two of the three subspecies of *Mab*, *Mab* subsp. *bolletii* and *Mab* subsp. *abscessus*, frequently develop macrolide resistance through mutations in the 23S rRNA (*rrl*) gene or induction of the erm(41) gene (3, 4, 40), resulting in the limited efficacy of macrolides. SOC has demonstrated limited efficacy against *Mab* in clinical and laboratory studies (25, 29, 30). In the search for safer and more effective therapeutic options, the efficacy of β-lactams, either in combination with another β-lactam or with BLIs, has been demonstrated in preclinical models and clinical investigations (12, 15, 16, 19, 20, 23, 25, 41–50).

The pace of new antibiotic development has slowed significantly in recent decades due to economic and scientific challenges. While β-lactams are extensively utilized for treating gram-negative bacteria, their potential against *Mycobacterium* remains underexplored. Currently, imipenem and cefoxitin are included in treatment guidelines for *Mab* infections (40); however, both agents have shown limited efficacy as monotherapies, with poor treatment outcomes or high failure rates even at elevated concentrations (Fig. S1). This underscores the critical need for novel β-lactams, such as next-generation carbapenems, to combat *Mycobacterium* infections.

The two carbapenem candidates, JDB/NA-1-157 and JDB/NA-1-208, share a similar chemical structure, differing only in their C5α substituents: JDB/NA-1-157 is a C5α-methyl analog, while JDB/NA-1-208 is a C5α-ethyl analog. Despite their structural similarity, these compounds exhibit markedly different antibacterial activities against *Mab*. JDB/NA-1-157 alone demonstrates significant bacterial killing even at lower concentrations, whereas JDB/NA-1-208 shows minimal activity as a single agent and requires combination with other β-lactams to exert an effect. This finding is intriguing and significant, as imipenem alone achieved only approximately a 1.5 log_10_ reduction in bacterial load (Fig. S1), suggesting that JDB/NA-1-157 may possess sufficient activity as a stand-alone therapeutic against *Mab*.

Interestingly, JDB/NA-1-157 is a substrate for Bla_Mab_, suggesting it may be hydrolyzed, whereas JDB/NA-1-208 appears resistant to Bla_Mab_-mediated hydrolysis. However, our kinetic analysis revealed that JDB/NA-1-157 has low catalytic efficiency as a substrate for Bla_Mab_, indicating it would be poorly hydrolyzed by this β-lactamase, with a *k*_cat_/*K*_*m*_ value notably lower than that of imipenem (20) (Table 3). Therefore, the disparity in bacterial killing is likely driven by differences in binding to peptidoglycan target receptors, including LDTs, PBPs, and D,D-carboxypeptidases, rather than by Bla_Mab_-mediated hydrolysis. JDB/NA-1-157 exhibited synergistic activity when combined with other β-lactams or durlobactam, whereas its combination with avibactam did not show significant synergy (only one- to twofold differences in MIC). These findings suggest that although JDB/NA-1-157 is a target of Bla_Mab_, its poor substrate profile may reduce the necessity for a combination BLI.

Notably, JDB/NA-1-208 lacks binding to PBP-lipo and D,D-carboxypeptidases compared to JDB/NA-1-157 (Fig. 6; Tables 2 and 4). These differences in target engagement likely explain the observed variations in antibacterial efficacy. This finding further supports the “target redundancy” hypothesis previously proposed by our group (15, 16, 20, 25, 51), highlighting the critical role of binding affinity and target specificity in determining antibacterial outcomes.

Compared to imipenem, which is known to be the most effective agent in *Mab* treatment, JDB/NA-1-157 demonstrated significant bacterial killing on its own, an effect not observed with imipenem alone (Fig. 3 to 5; Fig. S1). JDB/NA-1-157 showed lower catalytic efficacy against Bla_Mab_ but demonstrated a high binding affinity to several key PBPs, including PBP-lipo, PonA2, and D,D-carboxypeptidase. Although JDB/NA-1-157 showed lower binding affinity for LDT1 and LDT2 than imipenem, it still bound effectively to these targets. Notably, JDB/NA-1-157 exhibited weak binding to LDT3–5, which is considered less critical for bacterial killing. This selective binding to key LDTs/PBPs and low catalytic efficacy to Bla_Mab_ may contribute to its potent bacterial killing activity compared to imipenem.

Our MS analysis of the acylation of LDTs and PBPs by two atypical carbapenems reveals that both compounds form covalent adducts by binding as intact molecules. Fragmentation events were also observed in specific interactions with LDTs, PBPs, and Bla_Mab_. JDB/NA-1-157 underwent fragmentation of the acyl-enzyme to a derivative with a mass change of −32 Da (Δ*m* = +carbapenem −32 Da) in interactions with Bla_Mab_ and PBPB. Both JDB/NA-1-157 and JDB/NA-1-208 generated smaller derivatives, including a cysteine adduct (Δ*m* = +88 Da for LDT1) and a distinct fragmentation product (Δ*m* = +36 Da for LDT2). For LDT1, both the intact carbapenem-bound acyl-enzyme (Δ*m* = +carbapenem) and the hydroxybutyryl acyl-enzyme (Δ*m* = +88 Da) were detected. Both carbapenems acylate LDT1 and subsequently undergo nonhydrolytic fragmentation of the acyl-enzyme, producing a smaller hydroxybutyryl-cysteine derivative (Δ*m* = +88 Da). Considering the typical ±5 Da error in MS measurements, these results align with the predicted hydroxybutyryl acyl-enzyme structure (*m* = +86 Da). This type of fragmentation has been previously reported for Ldt_Mt2_ interacting with penem, faropenem, and C5α-substituted carbapenems (31, 34, 35) and for Ldt_Mab2_ interacting with sulopenem (16).

JDB/NA-1-157 adopts conformations where the carbonyl group is oriented toward the oxyanion hole and stabilized in these productive conformations. The newly introduced methyl group at C5α maintains a consistent π-methyl interaction with the indole ring of W105, irrespective of the W105 rotamer conformation (up or down). In contrast, both imipenem and meropenem prefer the conformation where the W105 rotamer is in the “up” position (*t-105*). The active site of LDT contains several conserved motifs known to interact with various compounds. Notably, the catalytic cysteine (C351) forms a covalent bond with C7 of carbapenems, and glycine (G350) plays a crucial role as part of the oxyanion hole. The variability of the active sites of LDT1–5 might cause differences in the preferential binding of JDB/NA-1-157. While all LDT1–5 variants contain a glycine residue at position 328 and share a conserved motif (SGI), LDT5, which does not bind JDB/NA-1-157, features an arginine and leucine (RGL) substitution at this position. This variation may impact interactions with the tail of JDB/NA-1-157. Methionine at position 300 is conserved across all LDT variants; however, the entrance to the active site displays greater variability. The tyrosine (Y105) present in LDT2 is replaced by phenylalanine in LDT5, which could disrupt the stabilizing interactions with the pyrroline ring of JDB-1-157, potentially affecting binding affinity. The tail of JDB/NA-1-157 appears more flexible, with the pyrrolidine ring derivative shifting to accommodate any conformational changes in the active site.

In summary, JDB/NA-1-157 demonstrated the most potent activity and shows potential as a single-agent option. JDB/NA-1-208, while less active alone, holds promise when combined with other β-lactam antibiotics, given its resistance to Bla_Mab_-mediated hydrolysis. Considering that most β-lactams, including imipenem, are susceptible to resistance mechanisms even when they are poor substrates for Bla_Mab_ (11, 12, 15, 17, 20, 25), BLIs may still be necessary to sustain efficacy in clinical settings. JDB/NA-1-208, when paired with BLIs or synergistic β-lactams, could therefore offer an additional viable therapeutic option. Future studies, including *in vitro* hollow fiber infection models, mouse models, and clinical trials, will be critical to validate the bacterial killing efficacy and therapeutic potential of these candidates.

## MATERIALS AND METHODS

### Bacterial strains, antibiotics, and reagents

We investigated the effect of C5α-substituted carbapenems (C5α-methyl, JDB/NA-1-157 or *10a* and C5α-ethyl, JDB/NA-1-208, or *10b*) against *Mab* strain ATCC 19977, which produces Bla_Mab_, alongside 18 clinical isolates obtained from de-identified patients. These clinical isolates were sourced from National Jewish Health, University Hospitals Cleveland Medical Center, and Cleveland MetroHealth. The strains were utilized for MIC assays and static drug concentration time-kill (SCTK) experiments. Two C5α-substituted carbapenems were designed and synthesized in the Buynak lab at SMU (31). The active forms of cefuroxime salts, imipenem, amoxicillin, sulbactam, and avibactam were purchased from AchemBlock, while durlobactam was provided by Innoviva Specialty Therapeutics. All β-lactams and BLIs were prepared in sterile distilled water and subsequently filter-sterilized using a 0.22 µm polyethersulfone syringe filter.

### Thermal stability

The stability of the two compounds in 7H9 broth was assessed at 30°C at drug concentrations of 10, 50, and 100 mg/L, with each condition tested in triplicate. Absorbance at 240 nm was measured every 2 h over a 24 h period using a BioTek Synergy2 multimode reader, with data analyzed via Gen5 software to monitor degradation or changes in stability. To evaluate the impact of thermal instability, MIC tests were performed using *Mab* ATCC 19977. Compounds were tested at time points of 0 h (no incubation) and 6, 24, and 48 h of incubation at 30°C. Imipenem and meropenem, known to be thermally unstable β-lactams, were included as positive controls. The MIC results were recorded after 48 h of incubation. Detailed MIC testing protocols were identical to those described below.

### MIC testing

MICs were determined in duplicate following Clinical Laboratory Standards Institute (52) guidelines using Middlebrook 7H9 broth supplemented with 10% (vol/vol) oleic acid-albumin-dextrose-catalase (OADC) and 0.05% (vol/vol) Tween 80, with an inoculum of 5 × 10⁵ CFU/mL. MIC testing of two C5α-substituted carbapenems was conducted both individually and in combination with cephalosporins (cefuroxime and ceftaroline), penicillins (amoxicillin), or BLIs (avibactam or durlobactam + sulbactam). Avibactam, durlobactam, cefuroxime, ceftaroline, and amoxicillin were fixed at concentrations of 4, 1, 2, 4, and 4 mg/L, respectively. Amoxicillin was combined with 4 mg/L avibactam, as it is highly susceptible to hydrolysis by Bla_Mab_ (12, 15, 25). The MIC was defined as the lowest antibiotic concentration that inhibited visible bacterial growth following 48 h of incubation at 30°C.

### SCTK assay

SCTK studies were conducted over 5 days in Middlebrook 7H9 broth supplemented with 10% (vol/vol) OADC, 0.2% (vol/vol) glycerol, and 0.05% (vol/vol) Tween 80 in duplicate. The efficacy of JDB/NA-1-157 and JDB/NA-208 was evaluated using *Mab* strain ATCC 19977 and three clinical isolates (Mab122, Mab686, and UH01) at an initial inoculum of 10^5.5^–10^6.3^ CFU/mL. These clinical isolates were carefully selected to include those with normal antibiotic activity (Mab122) (16) as well as an isolate exhibiting imipenem resistance (UH01). Bacterial killing was assessed across a wide range of concentrations for JDB/NA-1-157 and JDB/NA-1-208 individually (0.0625–64.0 mg/L).

For the combination study, the concentrations of JDB/NA-1-157 and JDB/NA-1-208 were selected based on MIC results and time-killing profiles of the single-drug study, while the concentrations of other β-lactams and BLIs were chosen based on clinically relevant levels, determined from the predicted average unbound steady-state concentrations (fC_ss,avg_) in plasma at clinically relevant doses (27). The drug concentrations used were JDB/NA-1-157, 1 mg/L; JDB/NA-1-208, 8 mg/L; imipenem, 12 mg/L; ceftaroline, 8 mg/L; cefuroxime, 8 mg/L; amoxicillin, 2 mg/L; avibactam, 4 mg/L; durlobactam, 12 mg/L; amikacin, 12 mg/L; clarithromycin, 0.3 mg/L; and cefoxitin, 7 mg/L. The SOC regimen was included for comparison of efficacy. On day 3, the broth was replaced with fresh broth containing the corresponding drug concentrations. For viable cell counts, 100 µL of undiluted or appropriately diluted samples was subcultured on Middlebrook 7H10 agar plates supplemented with 1% (vol/vol) OADC, 0.2% glycerol, and 0.05% (vol/vol) Tween 80. All samples were washed twice with sterile 0.9% saline prior to serial dilution or plating.

### Cloning and purification of LDTs and PBPs

Truncated sequences of Bla_Mab_ (Δ1–30 bla_Mab_), LDT1–5, DDC, PBP B, PonA2, and PBP-lipo (Δ1–41) were synthesized by Celtek Biosciences (Franklin, TN) and cloned into the pET28(a)+ vector. Purification of LDTs and PBPs was carried out as previously described (15, 16, 20). Briefly, clones were transformed into *Escherichia coli* BL21 (DE3), and protein expression was induced with 0.25 mM isopropyl β-d-1-thiogalactopyranoside. Cell pellets were resuspended in buffer containing 50 mM Tris (pH 8.0), 400 mM sodium chloride, and 1 mM Tris(2-carboxyethyl)phosphine hydrochloride and lysed by sonication. After centrifugation, the supernatant was purified using a His Prep FF column and eluted with 500 mM imidazole. While the His tags of the Bla_Mab_ protein were removed using thrombin, the His tags of LDTs and PBPs were cleaved with TEV protease, except for PBP-lipo and LDT5, due to their instability.

### Mass spectrometric analysis

The purified proteins were incubated at room temperature with JDB/NA-1-157 and JDB/NA-1-208 at a molar ratio of 1:10 for 2 h. A quadrupole time-of-flight mass spectrometer (Waters Synapt-G2-Si) with electrospray ionization and a Waters Acquity H-Class UPLC system were used to analyze the samples. The liquid chromatography conditions were as follows: a BEH C18 column (1.7 µm, 2.1 × 50 mm) and a mobile phase consisting of 0.1% formic acid in water and 0.1% formic acid in acetonitrile, with a gradient elution from 10% to 90% acetonitrile over 11 min at a flow rate of 0.5 mL/min. The MS settings were optimized with a capillary voltage of 3 kV, a sampling cone voltage of 35 V, and source and desolvation temperatures of 100°C and 500°C, respectively. Gas flow rates were 100 L/h for the cone and 800 L/h for desolvation. The mass accuracy was maintained at ±5 Da.

### Bocillin-FL binding assay

The binding of JDB/NA-1-157 and JDB/NA-1-208 to PBP-lipo was assessed using a Bocillin-FL competitive assay as previously described (25). Briefly, purified PBP-lipo and the compounds (at a 1:10 molar ratio) were incubated in a buffer containing 10 mM sodium phosphate (pH 7.4) and 150 mM NaCl for 2 h at room temperature, followed by additional incubation with 1 µM Bocillin FL for 4 h at 37°C. Samples were boiled and analyzed by SDS-PAGE. The PBP-lipo:Bocilli complexes were detected via fluorescence at 488 nm using an Azure 300 gel imaging system. Band intensity was quantified using GelAnalyzer software.

### Steady-state kinetics

Kinetic inhibition studies of JDB/NA-1-157 against Bla_Mab_ were conducted using an Agilent 8453 diode array spectrophotometer (Agilent Technologies, Inc., Santa Clara, CA) in 100 mM 2-(N-morpholino)ethanesulfonic acid (MES) buffer (pH 6.4) at room temperature. The reaction mechanism is depicted in equation 1:


(1) $$E+I\overset{k_{\text{1}}}{\underset{k_{-\text{1}}}{⇌}}E:I\overset{k_{\text{2}}}{\underset{k_{-\text{2}}}{⇌}}E-I,$$

where *E* represents the enzyme and *I* denotes the inhibitor. Kinetic assays were performed with a final concentration of nitrocefin (NCF) at 5× its Michaelis constant (*K*_*m*_; *K*_*m*_^NCF^ = 22 µM) and 7.0 nM Bla_Mab_, with JDB/NA-1-157 concentrations ranging from 0 to 0.05  µM. Catalytic efficiency (*k*_cat_/*K*_*m*_) was determined by monitoring the activity of Bla_Mab_ in the presence of increasing concentrations of JDB/NA-1-157.

Steady-state kinetic studies with JDB/NA-1-157 and JDB/NA-1-208 were performed using purified LDTs and PBPs, including LDT1–5, PBP-B, PBP-lipo, and D,D-carboxypeptidase. The assays were conducted on a BioTek Synergy2 multimode reader equipped with Gen5 software. To determine the Michaelis constant (*K*_*m*_^NCF^) for each LDT and PBP, a wide range of NCF concentrations was tested, while the enzyme concentrations were fixed. Absorbance changes at 482 nm were monitored over 10 min. Steady-state kinetic parameters (*V*_max_ and *K*_*m*_) were derived by fitting the experimental data to the Henri–Michaelis–Menten equation using nonlinear least-squares regression in GraphPad Prism version 9. The reaction velocity (*v*) at a given substrate concentration ([*S*]) was calculated using equation 2:


(2) $$v=\frac{V_{max}\times \left[S\right]}{K_{m}+\left[S\right]}.$$

The apparent inhibition constants (*K*_i,app_) for JDB/NA-1-157 and JDB/NA-1-208 were determined under the following conditions: an NCF concentration of 5 × *K*_*m*_, fixed concentrations of LDT or PBP enzymes, and varying concentrations of JDB/NA-1-157 and JDB/NA-1-208. Absorbance changes at 482 nm were monitored over a 30 min period for each inhibitor concentration. The data were linearized using a Dixon plot, which involves plotting the reciprocal of the absorbance change (1/Δ*A*) against the concentrations of JDB/NA-1-157 or JDB/NA-1-208. Apparent *K*_i,app_ values were derived from the intersection points on the Dixon plot and were subsequently corrected for substrate concentration and enzyme affinity using equation 3:


(3) $$K_{i,app}=\frac{observed\;K_{i,app}}{1+\left[NCF\right]/K_{m}^{\;\;\;NCF}}.$$

### Molecular docking and simulation

The crystal structures of Bla_Mab_ (PDB #4YFM) and LDT2_Mab (PDB #5UWV) were used for molecular docking. The missing parts of the LDT2 structure were reconstructed using the SWISS-MODEL homology modeling server, as previously described (20). The structures were prepared for docking using DS2020 (Discovery Studio Client 2020; *Dassault Systèmes* BIOVIA, San Diego) modeling software. The crystallographic waters were removed, and the structure was further minimized using the conjugate gradient method, with a root mean square (RMS) gradient of 0.001 kcal/(mol × Å). Generalized Born with a simple switching solvation model was used, and long-range electrostatics were treated using a particle mesh Ewald method with periodic boundary condition. The SHAKE algorithm was applied. The intact JDB-1-157 and acylated JDB-1-157, IPM, and MEM were built and docked into the active site of Bla_Mab_ or LDT2 enzyme. LibDock protocol, a high-throughput algorithm, was used for docking ligands into the active site of the enzyme (53–55). In docking this protocol, ligand conformations are aligned to polar and apolar receptor interaction sites (hot spots), and the best scoring poses are reported. Since some of the output poses may have hydrogen atoms near the receptor, a CHARMm minimization step was enabled to optimize the docked poses. Furthermore, the generated poses were analyzed, and the best ranked based on the scoring score and the minimum distance from carbonyl C7 and catalytic S70 (or C351) were used to create the Michaelis–Menten and acyl-enzyme complexes. The complexes were further minimized to assess the stability of the systems.

## SUPPLEMENTAL MATERIAL

Supplemental material aac.00170-25-s0001.docx Fig. S1 and S2; Tables S1 and S2.

ASM does not own the copyrights to Supplemental Material that may be linked to, or accessed through, an article. The authors have granted ASM a non-exclusive, world-wide license to publish the Supplemental Material files. Please contact the corresponding author directly for reuse.
