# Supplementary material for: Novel C5α-substituted carbapenems enhance Mycobacterium abscessus killing via selective target binding and reduced hydrolysis by BlaMab
Source: Antimicrob Agents Chemother. 2025 Jun 17;69(8):e00170-25. doi: 10.1128/aac.00170-25 (PMC12326981; doi:10.1128/aac.00170-25)
Supplement: Supplemental material — Fig. S1 and S2; Tables S1 and S2. [file aac.00170-25-s0001.docx]

**Discussion**

We evaluated the thermal stability of JDB/NA-1-157, JDB/NA-1-208, and the clinically used carbapenems, imipenem and meropenem. Given that carbapenems undergo significant thermal degradation,[^1^](#_ENREF_1) we also performed MIC testing to assess the impact of degradation and minimize experimental error. Imipenem degrades most rapidly during the initial incubation period, with approximately 25% degradation within the first 6 h and an additional 25% by 24 h (**Fig. 2** and **Fig. S2**). However, while the MIC of imipenem remained unchanged between 'no incubation' and '6h incubation' (1 mg/L), it increased 32-fold after 24 h incubation (32 mg/L), despite similar degradation levels between 0–6 and 6–24 hours. This discrepancy can be explained by several factors.

First, although imipenem degradation occurs within the initial 6h incubation, it remains below two-fold, which is insufficient to affect MIC values given the two-fold dilution method. Second, the initial imipenem concentration plays a key role in MIC determination. In the 0h and 6h groups, the initial concentration remains high, maintaining antimicrobial activity. However, in the 24h and 48h groups, prolonged degradation results in significantly lower initial exposure, leading to increased MIC values. Third, differences in total incubation time may influence MIC results. The 0h and 6h groups undergo MIC testing for 48 and 54 hh, respectively, a minor difference comparable to the *Mab* doubling time. In contrast, the 24h and 48h groups have extended incubation times of 72 and 92 h, which may contribute to the observed MIC increase.

**Figure S1:** Time-kill curves of imipenem as monotherapy

**
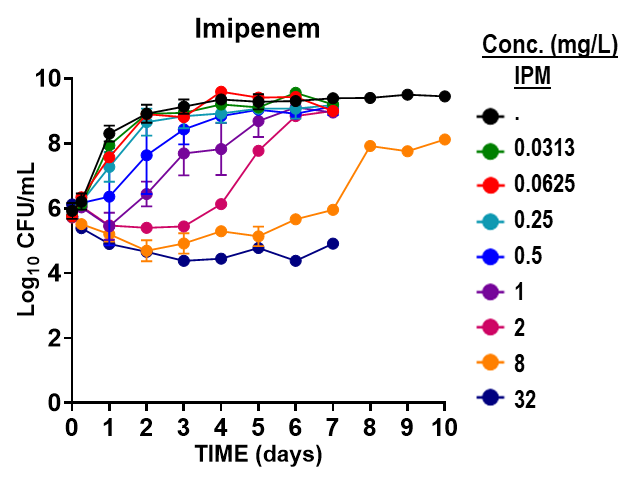
**

**Figure S2:** Thermal stability of imipenem (IPM), meropenem (MEM), JDB/NA-1-157, and JDB/NA-1-208 was evaluated at different concentrations (50 mg/L (A) and 10 mg/L (B)) in 7H9 broth at 30 °C over 24 h. JDB/NA-1-208 demonstrated the highest thermal stability, with approximately 10% degradation observed after 24 h. JDB/NA-1-157 exhibited 16% degradation over the same period.

**
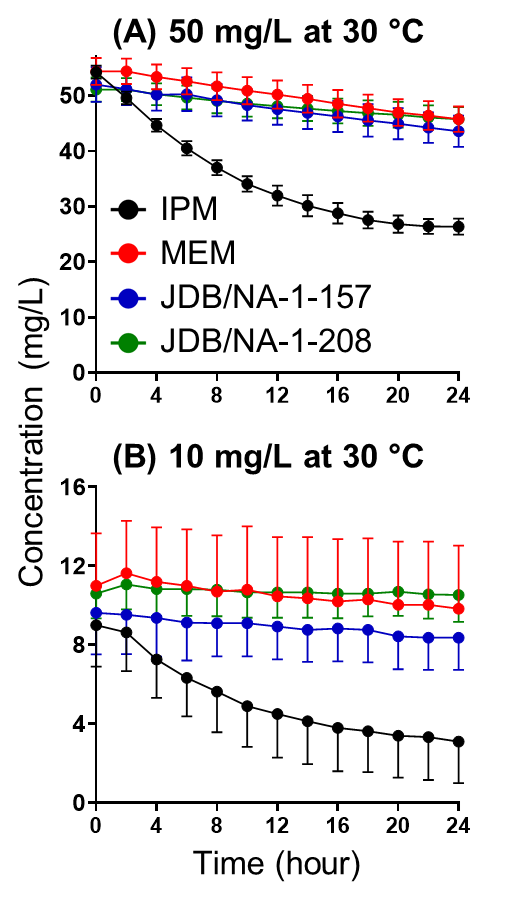
**

**Table S1:** Degradation percentage (%) of imipenem (IPM), meropenem (MEM), JDB/NA-1-157, and JDB/NA-1-208 in 7H9 broth in 24 h at 30 °C

|  | **100 mg/L** | **50 mg/L** | **10 mg/L** |
| --- | --- | --- | --- |
| **IPM** | **49.3** | **51.4** | **67.8** |
| **MEM** | **16.1** | **15.8** | **11.7** |
| **JDB/NA-1-157** | **16.6** | **16.2** | **11.9** |
| **JDB/NA-1-208** | **8.2** | **10.5** | **0.6** |

**Table S2:** Effect of drug instability on MIC testing. MIC (mg/L) values of imipenem (IPM), meropenem (MEM), JDB/NA-1-157, and JDB/NA-1-208 were determined against *Mab* ATCC 19977. MIC tests were conducted using both non-incubated drugs and drugs incubated in 7H9 broth for 6, 24, and 48 hours. Results were recorded after 2 days.

| (mg/L) | IPM | MEM | JDB/NA-1-157 | JDB/NA-1-208 |
| --- | --- | --- | --- | --- |
| **No incubation** | 1 | 4 | 0.25 | 16 |
| **6h incubation** | 1 | 4 | 0.25 | 16 |
| **24h incubation** | 32 | 16 | 2 | 32 |
| **48h incubation** | >64 | 16 | 2 | 32 |

**References**

1. **Zhou J, Qian Y, Lang Y, Zhang Y, Tao X, Moya B, Sayed ARM, Landersdorfer CB, Shin E, Werkman C, Smith NM, Kim TH, Kumaraswamy M, Shin BS, Tsuji BT, Bonomo RA, Lee RE, and Bulitta JB.** Comprehensive stability analysis of 13 beta-lactams and beta-lactamase inhibitors in in vitro media, and novel supplement dosing strategy to mitigate thermal drug degradation. Antimicrob Agents Chemother **2024**. 68**:**e0139923. PMC10916406
